# Supplementary material for: Tracking country-level mitigation progress using NGHGI-consistent carbon budgets
Source: Nat Commun. 2026 Feb 13;17:1494. doi: 10.1038/s41467-026-69078-9 (PMC12905252; doi:10.1038/s41467-026-69078-9)
Supplement: Supplementary file 1 — Supplementary Information [file 41467_2026_69078_MOESM1_ESM.pdf]

Supplementary Information for: Tracking Country-level Mitigation Progress Using NGHGI-Consistent Carbon Budgets

Konstantin Weber[1], **Cyril Brunner**[1] **and Reto Knutti**[1]
[1] Institute for Atmosphere and Climate Science ETH Zurich, Zurich, Switzerland

E-mail: konstantin.weber@env.ethz.ch

1 Supplementary Note 1: Potential overestimation of the NGHGI-consistent global RCB

There is reason to assume that the correction applied to the global IPCC-based RCB is underestimated: In the reanalysis performed by Gidden et al.[1] the difference between LULUCF CO_2 emissions reported by bookkeeping models (BM)[2] and in National Greenhouse Gas Inventories (NGHGI)[3,4] is underestimated in the period from 1990 to 2023. Hence, we judge an underestimation of the future difference until net zero CO_2 also to be likely (Supplementary Fig. 1). The mismatch amounts to around 3 $GtCO_2$/year (Supplementary Fig. 2) and may be related to a too-high CO_2 fertilization effect in the BM OSCAR[5]. Taking this into account, the NGHGI-consistent global RCB for 1.5 °C (50%) is further reduced by the order of 10 to 100 $GtCO_2$. In addition, current projections for the growth of the aviation sector (e.g.,[6]) and aviation sector-specific scenarios[7] indicate higher future CO_2 emissions than found in global 1.5 °C compatible scenarios. This could signify already an exceedance of the 1.5 °C compatible CO_2 emissions reported in NGHGIs as of 1 January 2025.

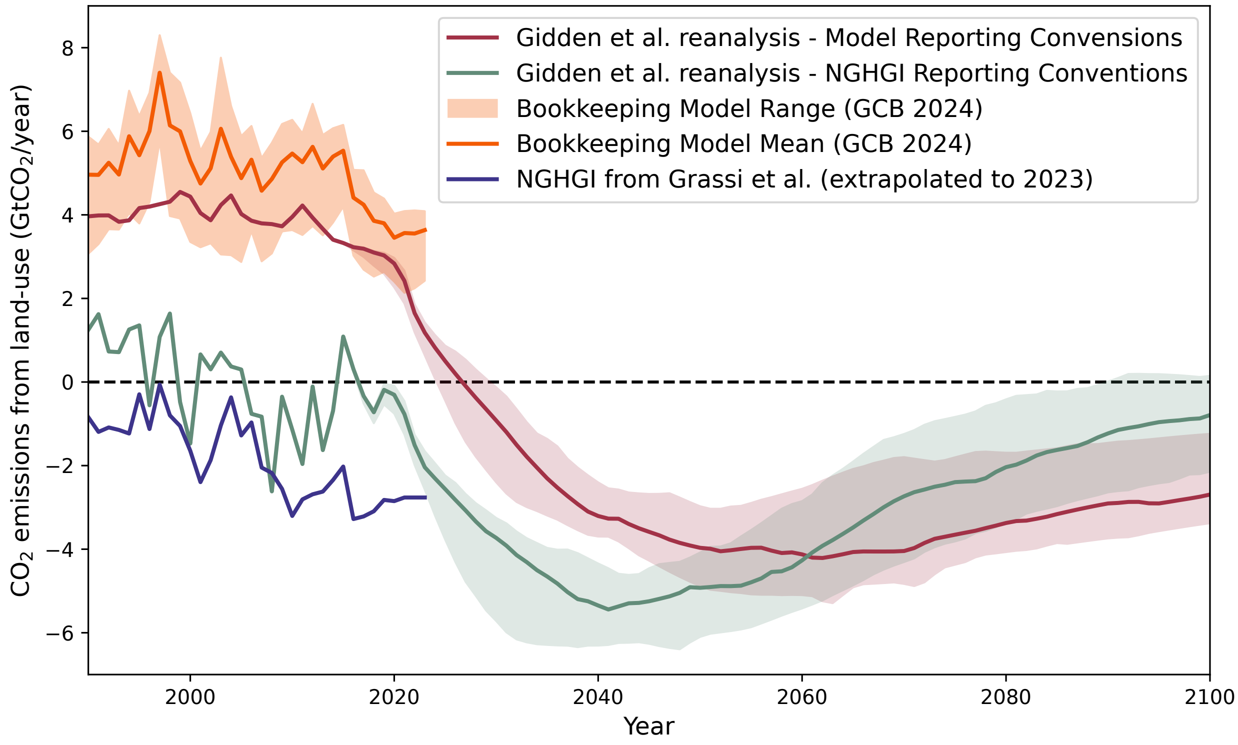

Supplementary Figure 1: **Comparison of LULUCF CO_2 emissions estimated from bookkeeping models and reported in NGHGIs.** The green and red lines extending to 2100 are estimated LULUCF CO_2 emissions[1] for AR6 scenarios that keep global warming to 1.5 °C with no or limited overshoot. The orange range indicates LULUCF CO_2 emissions from four bookkeeping models[2], and the purple line shows aggregated NGHGI reported emissions[4].

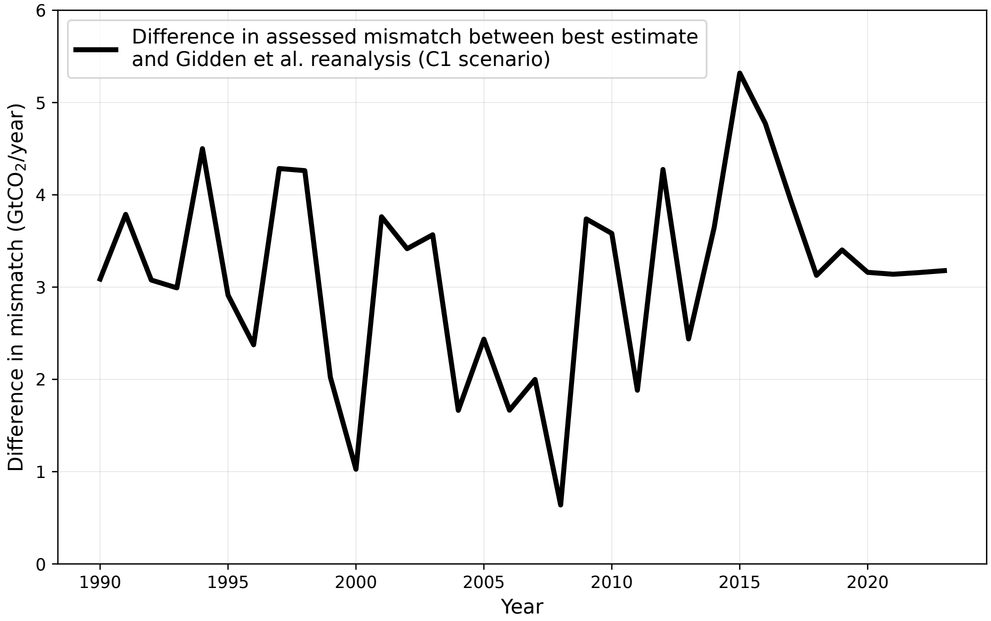

Supplementary Figure 2: **Difference in the model versus NGHGI reported LULUCF CO_2 emissions mismatch between what has been reported and the reanalysis performed by Gidden et al.[1].**

2 Supplementary Note 2: Change of interpretation when allocating a negative global RCB

Some allocation approaches assign a larger share of the global RCB to countries with higher emissions (e.g., grandfathering and Bretschger burden sharing). While the global RCB remains positive, this benefits high-emitting countries. By contrast, capacity-based approaches reduce the allocated share with increasing GDP per capita, reducing the share of the global RCB for wealthier nations. However, once a global temperature limit is overshot and the global RCB becomes negative, these interpretations invert: Approaches that previously penalized wealthy countries now advantage them, and vice versa. Caution is therefore required when applying the same operationalization principles once the global RCB switches sign.

3 Supplementary Note 3: The use of consumption-based emissions

The IPCC guidelines for national greenhouse gas inventories specify that "National inventories include greenhouse gas emissions and removals taking place within national territory and offshore areas over which the country has jurisdiction"[8], making clear that under the UNFCCC process reporting is based on territorial emissions. Likewise, national climate strategies and NDCs exclusively cover territorial emissions. An alternative perspective is provided by consumption-based accounting, which adjusts territorial CO_2 emissions for the carbon intensity of imported and exported goods (data provided, for example, in the Global Carbon Budget 2024[9]). This approach has been discussed in the literature as a way of attributing emissions to countries in line with their consumption patterns, and thus as a potential basis for assessing historical responsibility[10–12]. Some scholars have argued that it better captures national responsibility for climate change (e.g.,[13]). Even some countries' updated NDCs[14] mention the necessity to consider consumption-based emissions in their reflection on fair shares. Calculating national RCBs that account for historical responsibility for consumption-based CO_2 emissions is therefore a complementary (and arguably necessary) perspective, even though they may not be directly comparable to NDCs, and NGHGIs continue to be based on territorial emissions.

4 Additional Figures

4.1 The global NGHGI-consistent RCB

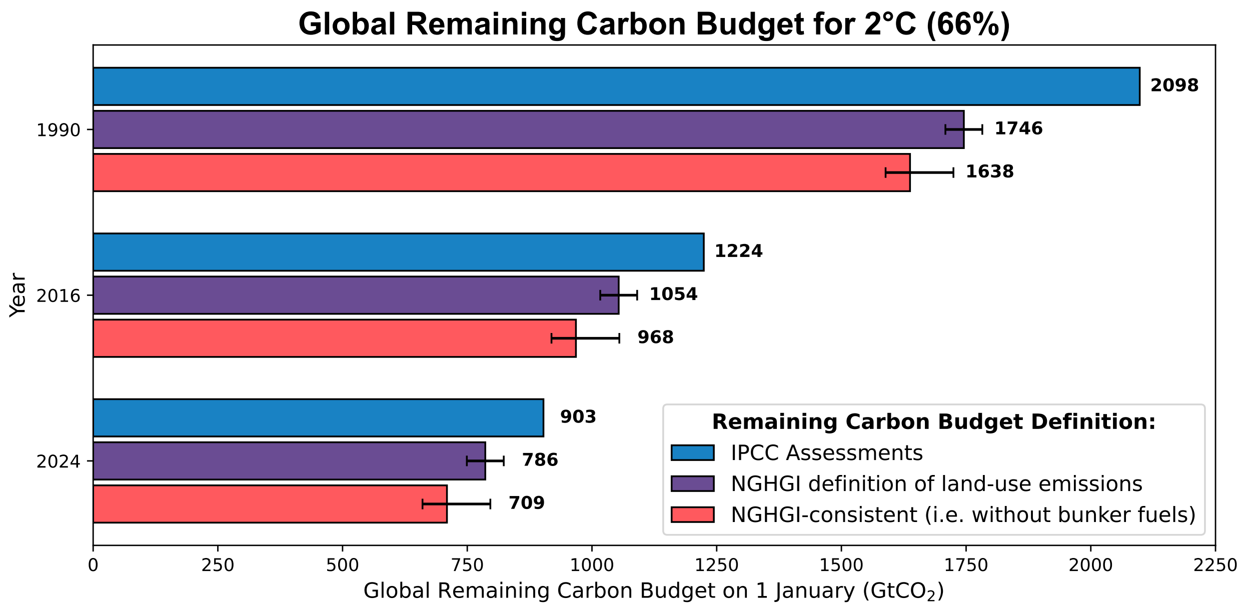

Supplementary Figure 3: **Comparison of alternative definitions of the 2 °C-compatible remaining carbon budget (RCB) for 1990, 2016 (the time of adoption of the Paris Agreement, and 2024.** This figure is equivalent to Fig. 1d of the main text, but corresponds to a higher temperature limit, which changes the IPCC-reported RCB and the correction terms applied. Error bars reflect uncertainty in correction terms. The lower (upper) bound is derived from the maximum (minimum) of assessed future bunker fuel emissions and the 95th (5th) percentile of C3 scenarios for the assessed mismatch in future LULUCF CO_2 emissions.

4.2 IPCC-based compared to NGHGI-consistent national RCBs for 2016

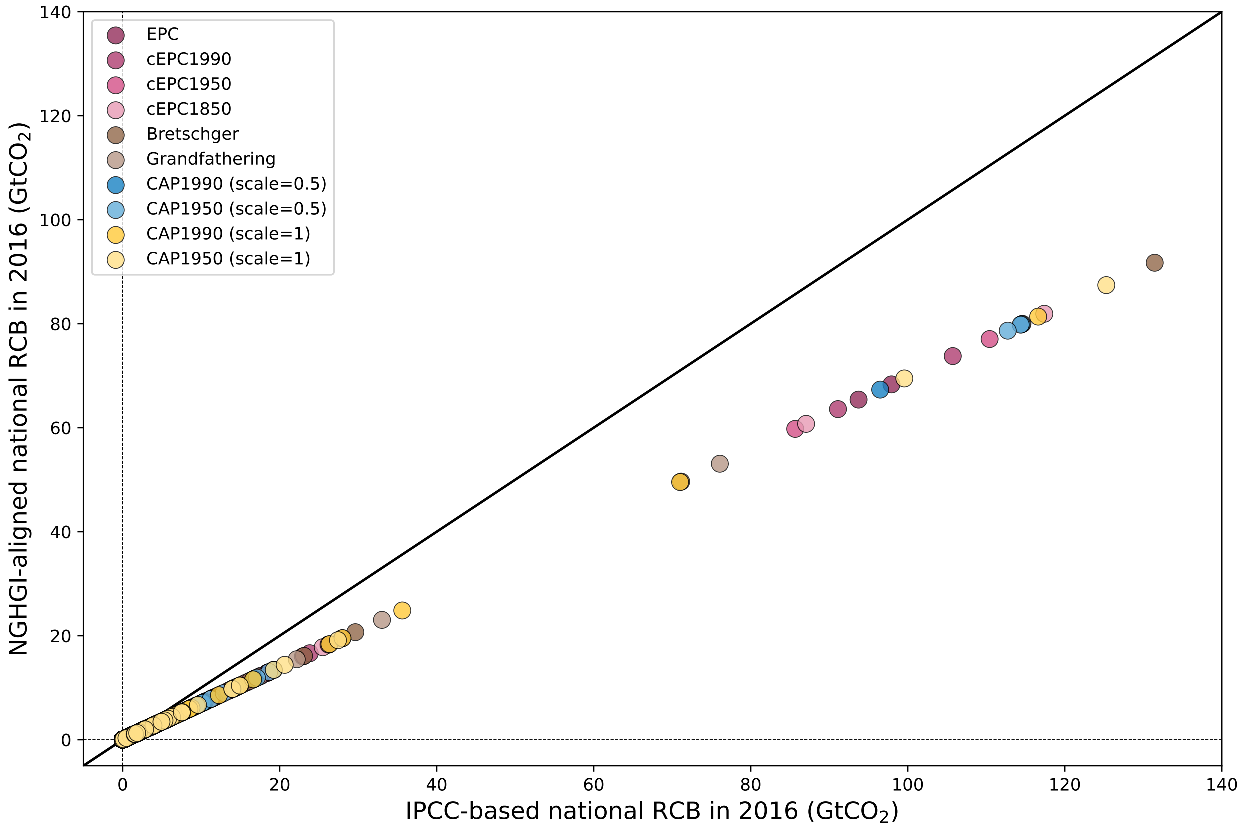

Supplementary Figure 4: **Effect of alignment for national RCBs that do not take historical responsibility into account.** Comparison of computed 1.5 °C-compatible NGHGI-consistent national RCBs with the corresponding IPCC-based national RCBs for the beginning of 2016 (approximate time of adoption of the Paris Agreement).

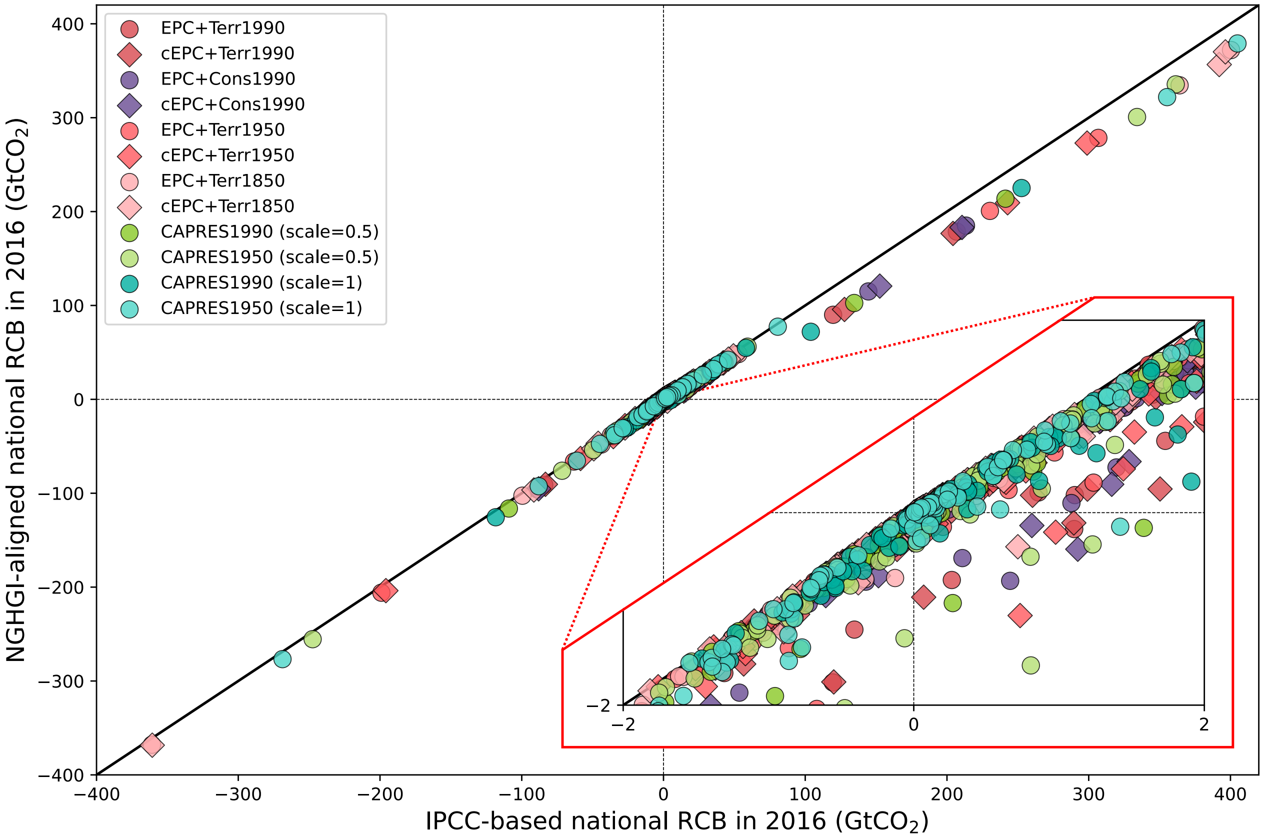

Supplementary Figure 5: **Effect of alignment for national RCBs that consider historical responsibility.** Comparison of computed 1.5 °C-compatible NGHGI-consistent national RCBs with the corresponding IPCC-based national RCBs for the beginning of 2016 (approximate time of adoption of the Paris Agreement).

4.3 Allocation of the NGHGI-consistent budget to countries

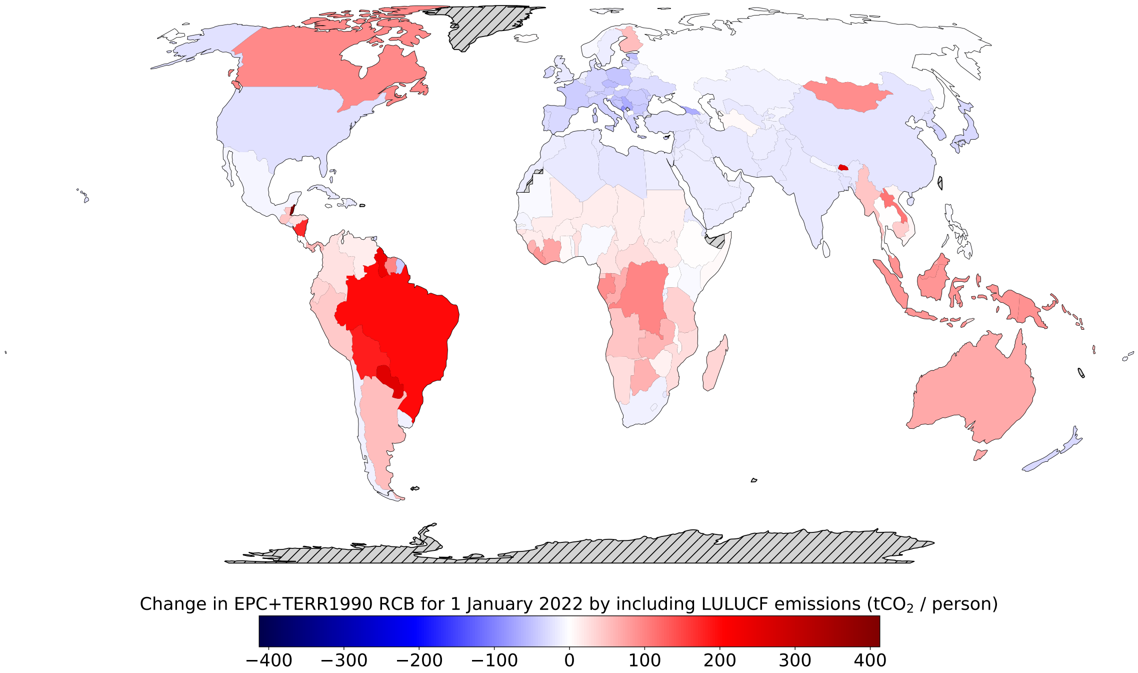

Supplementary Figure 6: **Carbon debt in 2022 associated with LULUCF CO_2 emissions since 1990.** This is obtained as the difference of the EPC+TERR1990 RCB calculated once with and once without inclusion of bookkeeping LULUCF CO_2 emissions. Map made with Natural Earth.

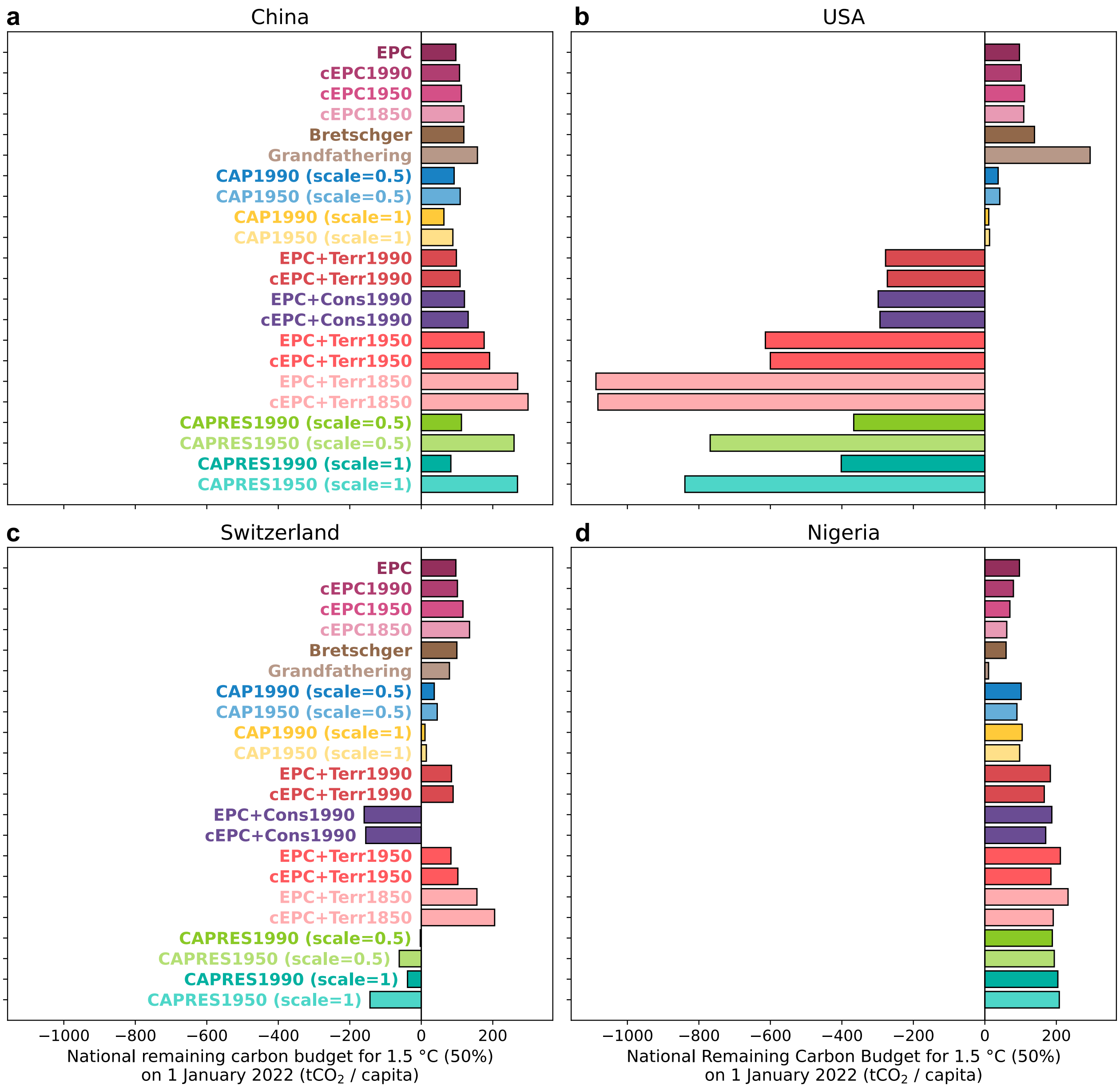

Supplementary Figure 7: **Per-capita 2 °C (66%) RCBs for China (a), the USA (b), Switzerland (c), and Nigeria (d) as of 1 January 2022, computed for a range of allocation principles and methods.** EPC denotes equal-per-capita approaches; Bretschger refers to Bretschger burden sharing; CAP stands for capacity-based approaches; EPC+TERR (EPC+CONS) considers historical responsibility for territorial (consumption-based) emissions, CAPRES takes capacity and historical responsibility into account. More details on the naming of allocation methods and their implementation are found in the Methods section *Distribution of the NGHGI-consistent global RCB across countries* of the main text.

4.4 Time-dependent national RCBs and exceedance of Paris Agreement temperature limits

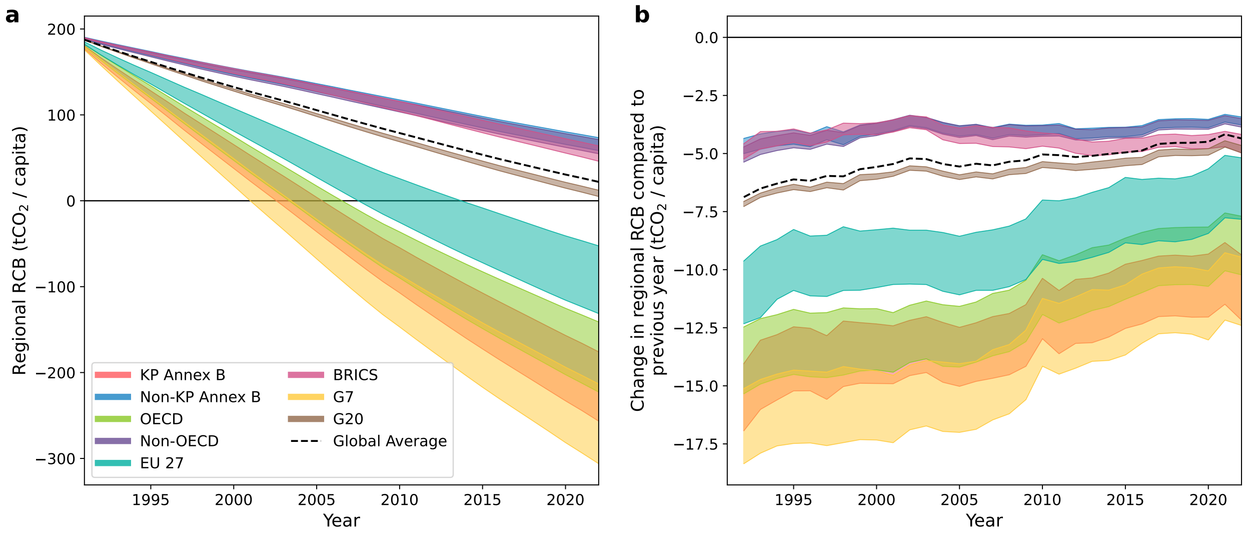

Supplementary Figure 8: **Temporal evolution of aggregated NGHGI-consistent RCBs for 1.5 °C (50%).**
a Per-capita RCBs for eight different country aggregations. **b** Corresponding changes in aggregated per-capita RCBs relative to the previous year.

Illustration of geographical distribution of exceedance

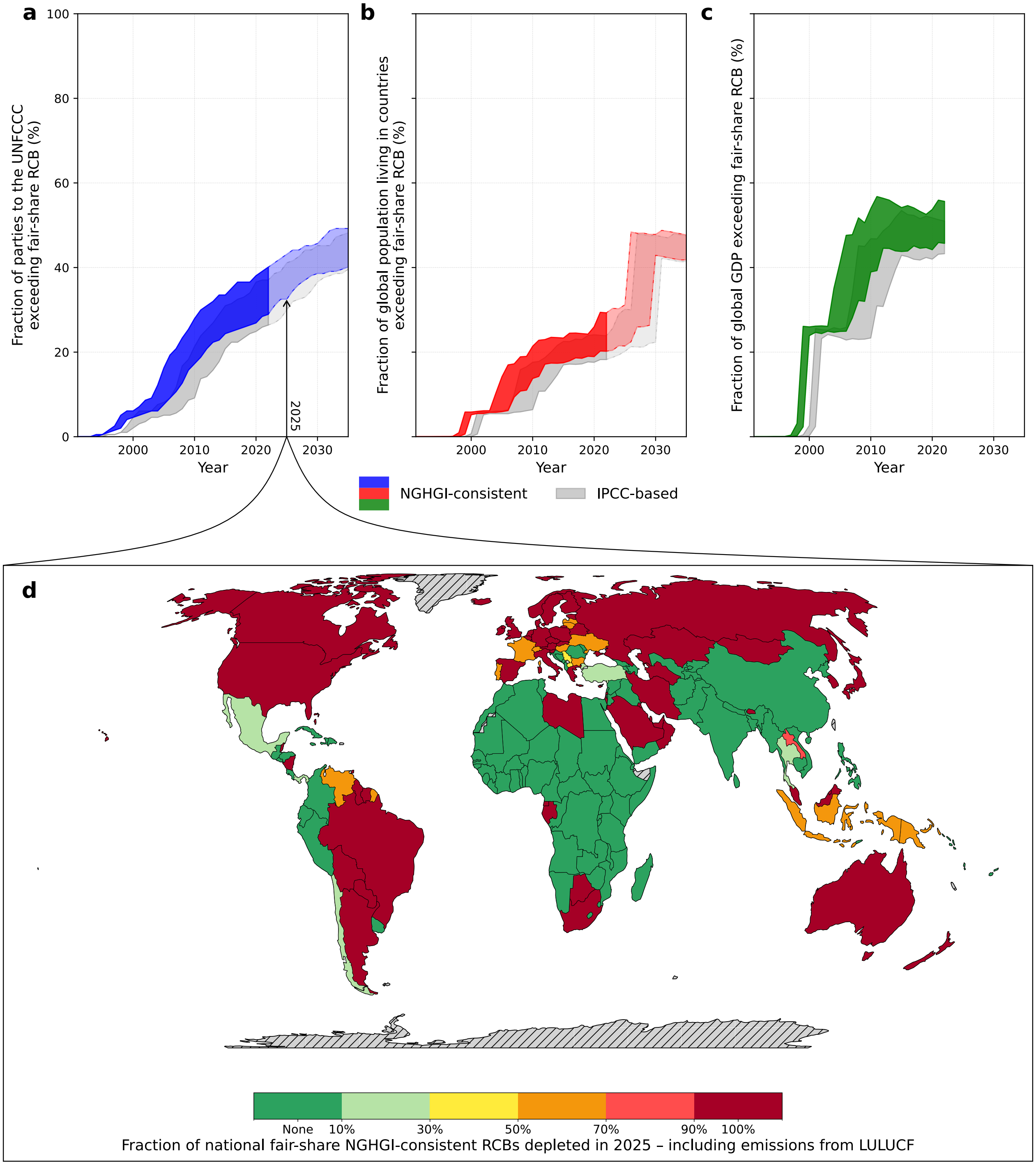

Supplementary Figure 9: **Fig. 4 of the main text (a, b, c) combined with a map (d) showing the estimated fraction of fair-share RCBs depleted as of 1 January 2025 for UNFCCC member states and other countries included in the provided dataset.** Map made with Natural Earth.

Fig. 4 of the main text for 2 °C (66%)

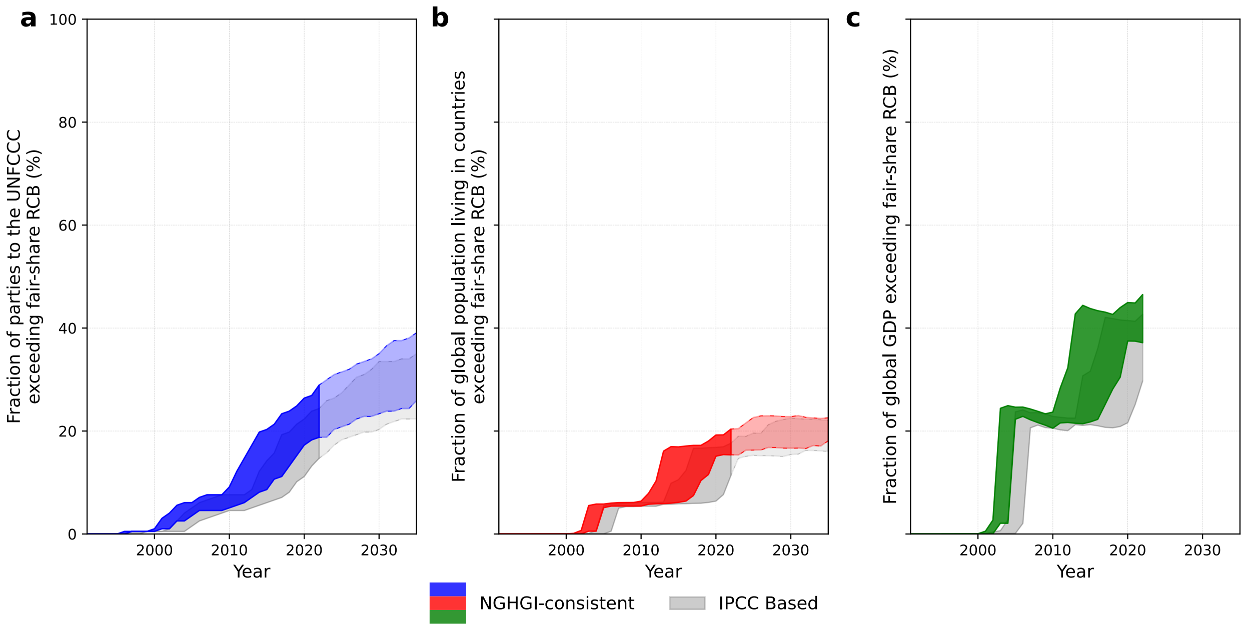

Supplementary Figure 10: **Fraction of UNFCCC countries exceeding their 2 °C (66%)-compatible fair-share RCB over time (a), alongside the share of global population (b) and GDP (c) they represent.** The shaded area indicates the range arising from the five selected allocation methods used to calculate national RCBs, each resulting in distinct timings of countries exceeding their fair-share of the global RCB. The gray shading corresponds to the results, if derived from an IPCC-based global RCB instead. Extrapolation to 2035 for UNFCCC parties and population is based on a quadratic extension, as described in the Methods section *Global Analysis* of the main text.

Dependence of RCBs on human development status

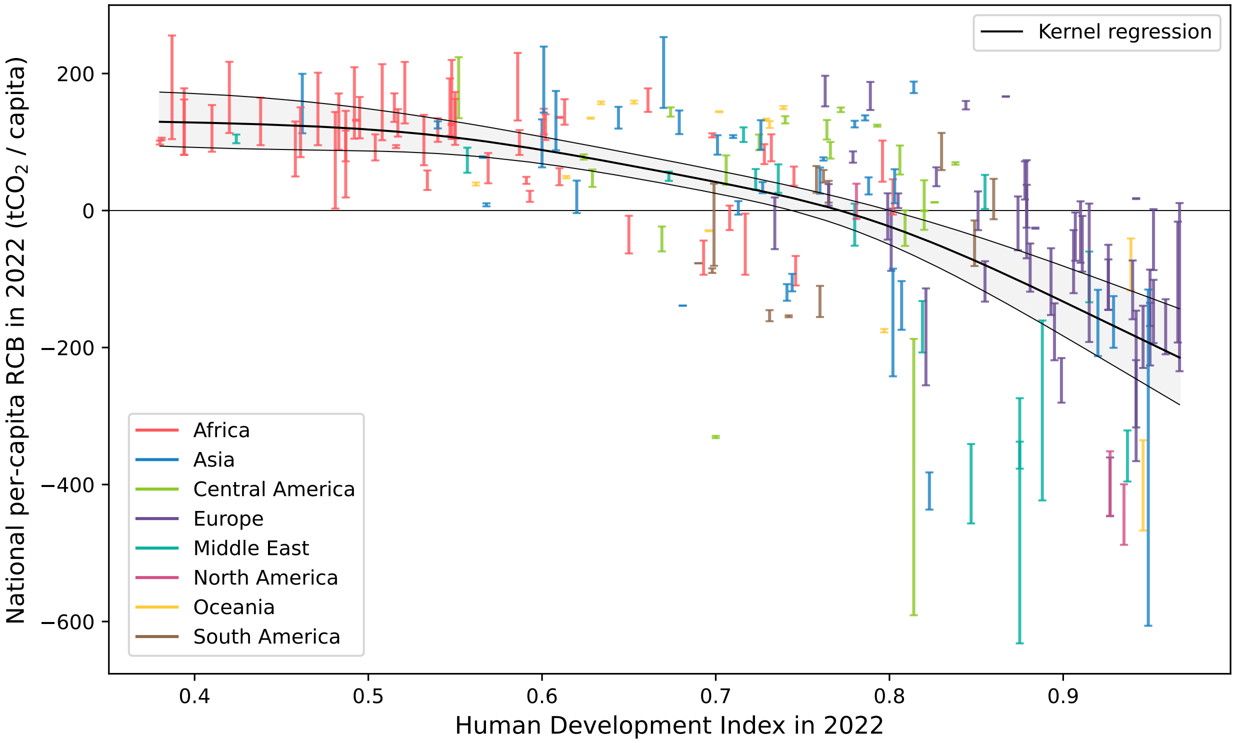

Supplementary Figure 11: **Dependence of the 2022 1.5 °C (50%) fair-share RCB on the Human Development Index[15] (HDI).** Ranges denote the full range of RCBs based on the five selected allocation methods that consider historical responsibility since 1990. Negative RCBs become predominant for HDI > 0.8, i.e., for countries with very high human development according to this metric.

4.5 The case of Switzerland's RCB and NDC

Fig. 6 of the main text, but considering historical responsibility only for fossil CO_2 emissions.

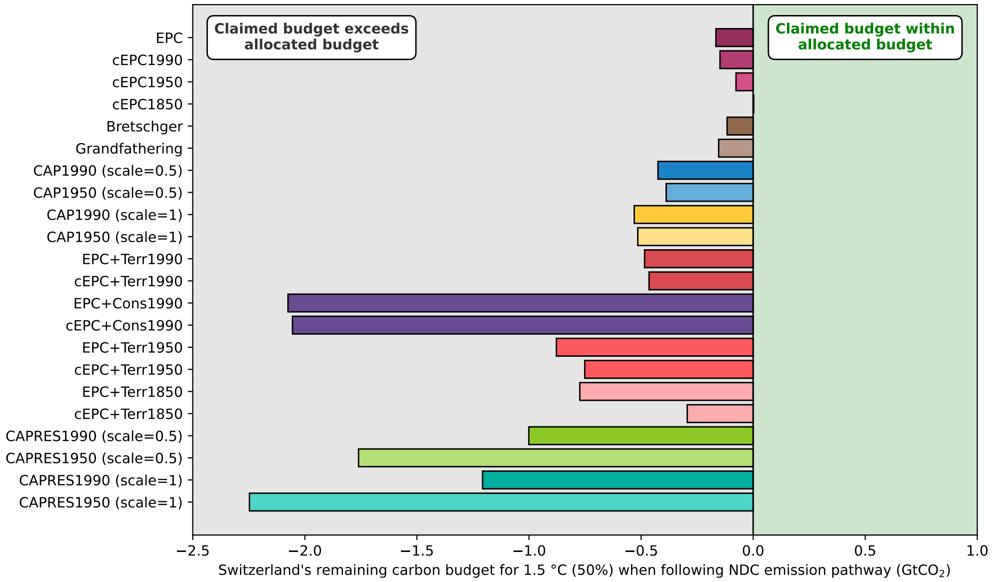

Supplementary Figure 12: **Comparison of Switzerland's 1.5 °C (50%) RCB to Switzerland's second NDC when considering historical responsibility for only fossil CO_2 emissions.** As in Fig. 6 of the main text, the differences between RCB estimates and future CO_2 emissions are shown for the start of 2016. For allocation approaches that do not take into account historical responsibility, the RCB estimates are the same as in Fig. 6 of the main text. Positive values suggest an allocated RCB larger than the post-2015 CO_2 emissions implied by Switzerland's NDC (green area to the right), while negative values indicate Switzerland overshooting its RCB (gray area to the left).

Fig. 6 of the main text, but for 2 °C (66%)

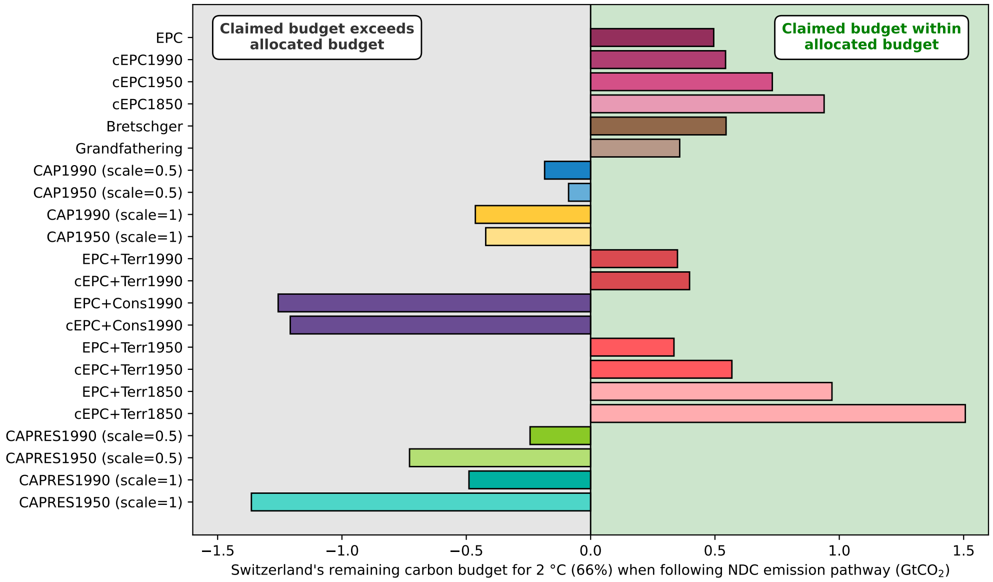

Supplementary Figure 13: **Comparison of Switzerland's 2 °C (66%) RCB to Switzerland's second NDC** Historical responsibility for LULUCF CO_2 emissions is considered here. Same illustration as in Fig. 6 of the main text, but for a different temperature limit.

Fig. 6 of the main text, but for 2 °C (66%) and considering historical responsibility only for fossil CO_2 emissions.

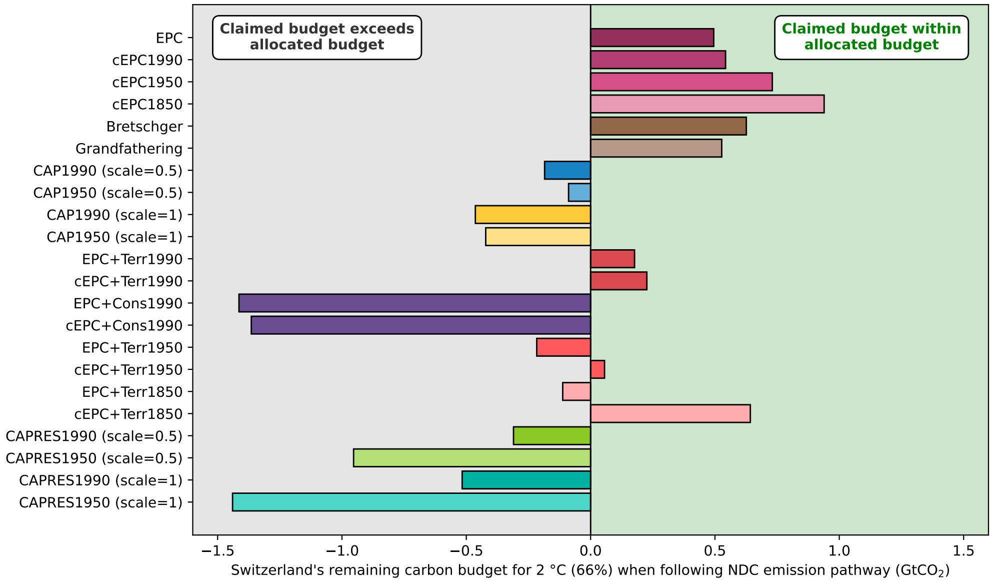

Supplementary Figure 14: **Comparison of Switzerland's 2 °C (66%) RCB to Switzerland's second NDC when considering historical responsibility for only fossil CO_2 emissions.** Same illustration as in Supplementary Fig. 10, but for a different temperature limit.

4.6 Historical responsibility for only fossil CO_2 emissions

Fig. 3 of the main text, but considering historical responsibility for only fossil CO_2 emissions

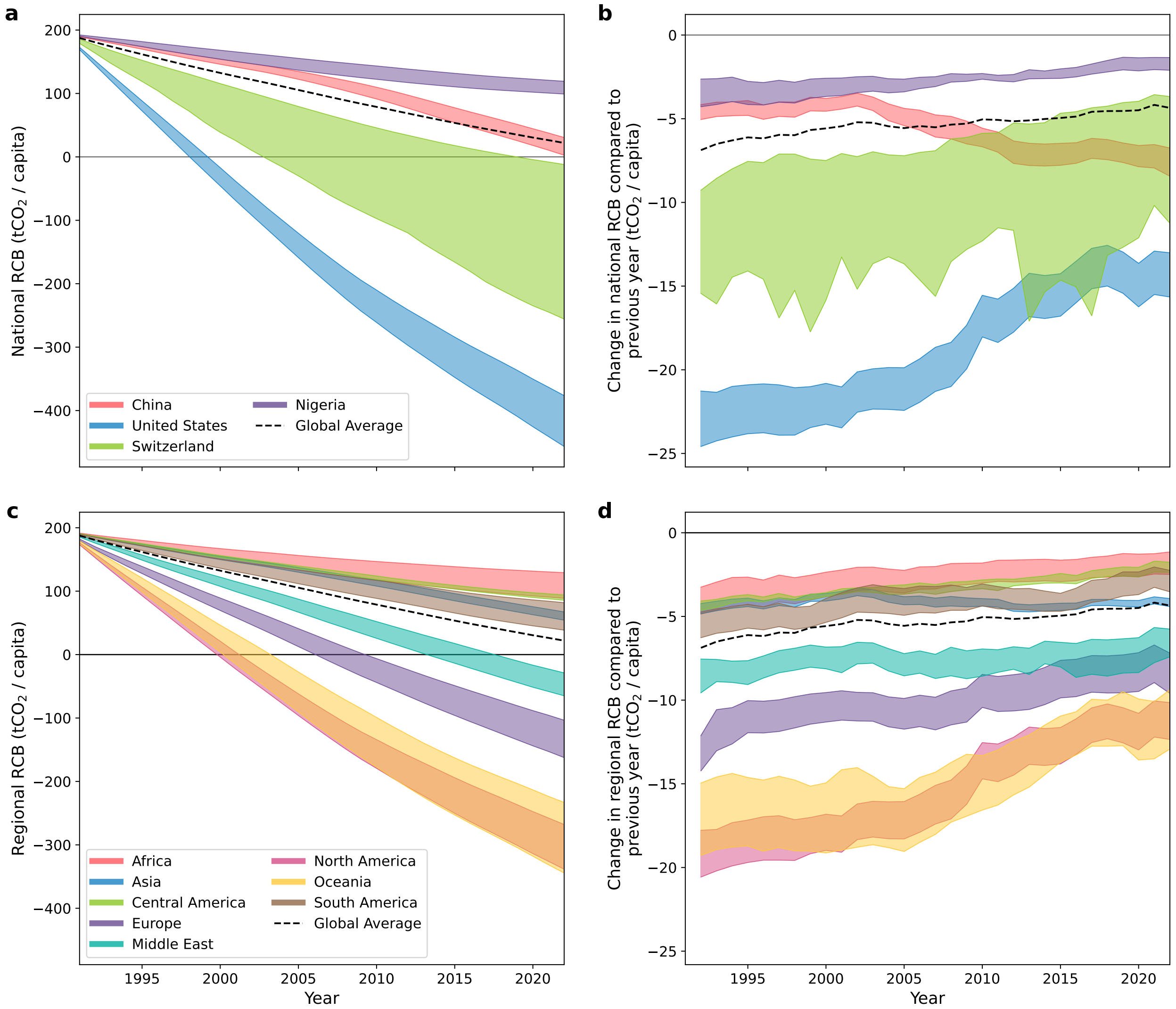

Supplementary Figure 15: **Temporal evolution of national and regional NGHGI-consistent RCBs for 1.5 °C (50%) considering historical responsibility for only fossil CO_2 emissions.** **a** National per-capita RCBs of China, the USA, Switzerland, and Nigeria since 1990 under a selection of fair-share allocation methods. **b** Corresponding changes in national per-capita RCBs relative to the previous year. **c** Per-capita RCBs for eight geographical regions. **d** Corresponding changes in regional per-capita RCBs relative to the previous year.

Fig. 4 of the main text, but considering historical responsibility for only fossil CO_2 emissions

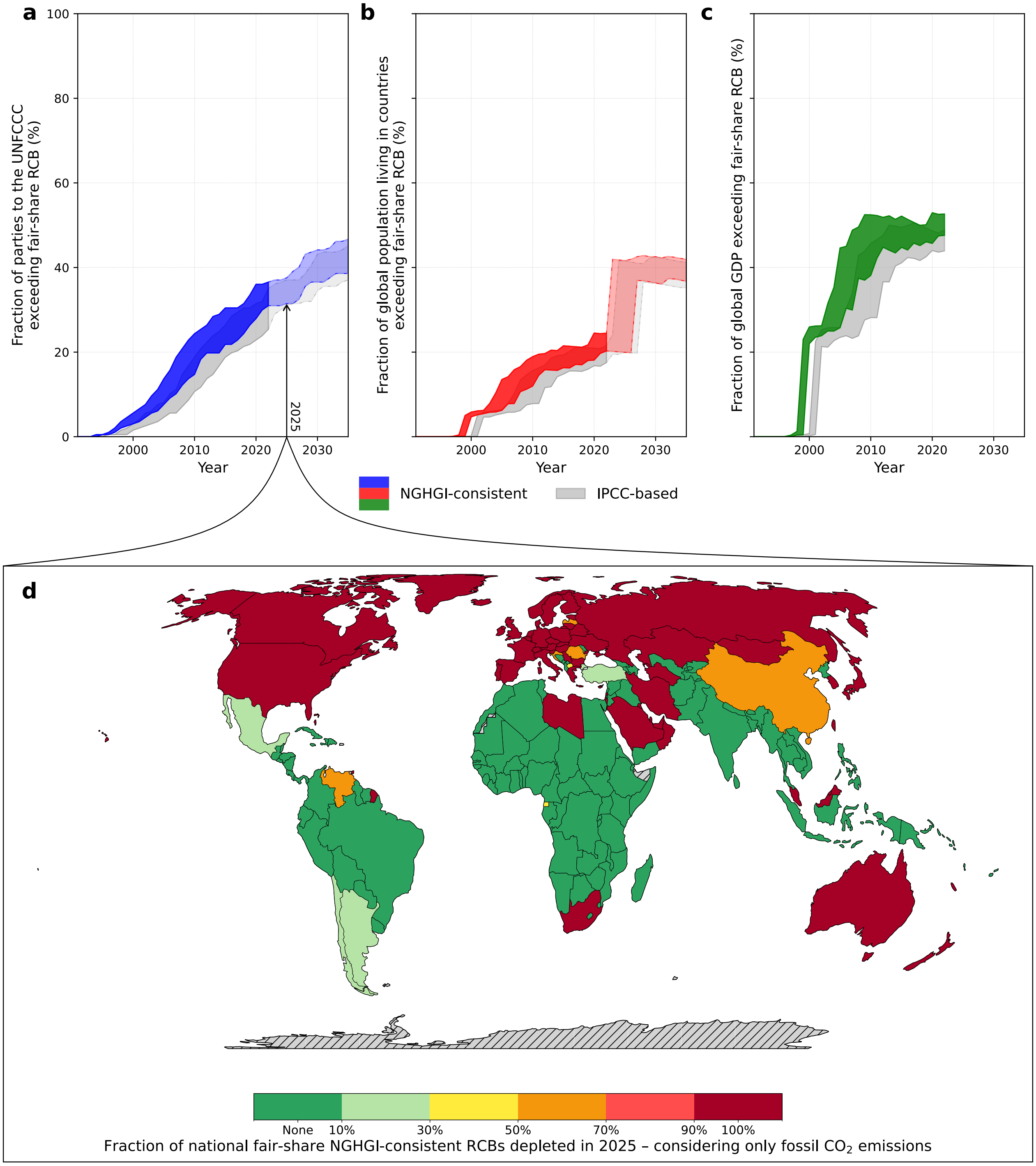

Supplementary Figure 16: **Fig. 4 of the main text (a, b, c), but considering historical responsibility only for fossil CO_2 emissions. The map (d) shows the estimated fraction of fair-share RCBs depleted as of 1 January 2025 for UNFCCC member states and other countries included in the provided dataset also when considering historical responsibility for only fossil CO_2 emissions.** Map made with Natural Earth.

Supplementary Fig. 11, but considering historical responsibility for only fossil CO_2 emissions

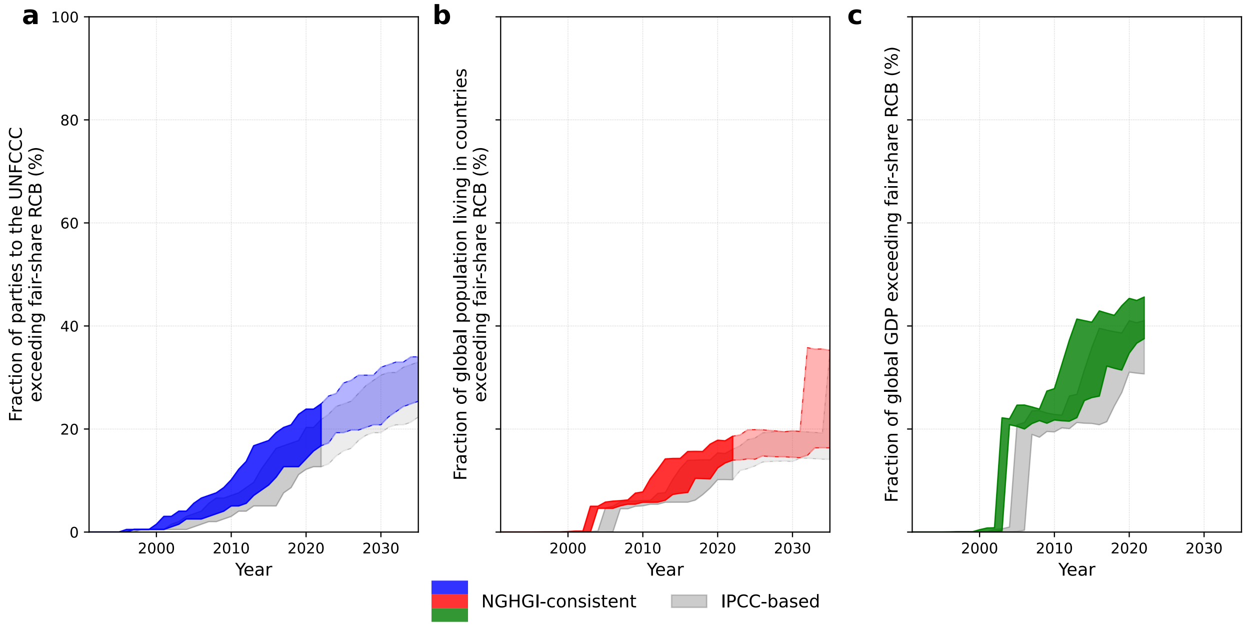

Supplementary Figure 17: **Fraction of UNFCCC countries exceeding their 2 °C (66%)-compatible fair-share RCB over time (a), alongside the share of global population (b) and GDP (c) they represent when considering historical responsibility for only fossil CO_2 emissions.** The shaded area indicates the range arising from the five selected allocation methods used to calculate national RCBs, each resulting in distinct timings of countries exceeding their fair-share of the global RCB. The gray shading corresponds to the results, if derived from an IPCC-based global RCB instead. Extrapolation to 2035 for UNFCCC parties and population is based on a quadratic extension, as described in the Methods section *Global Analysis* of the main text.

4.7 Future CO_2 emissions from bunker fuels

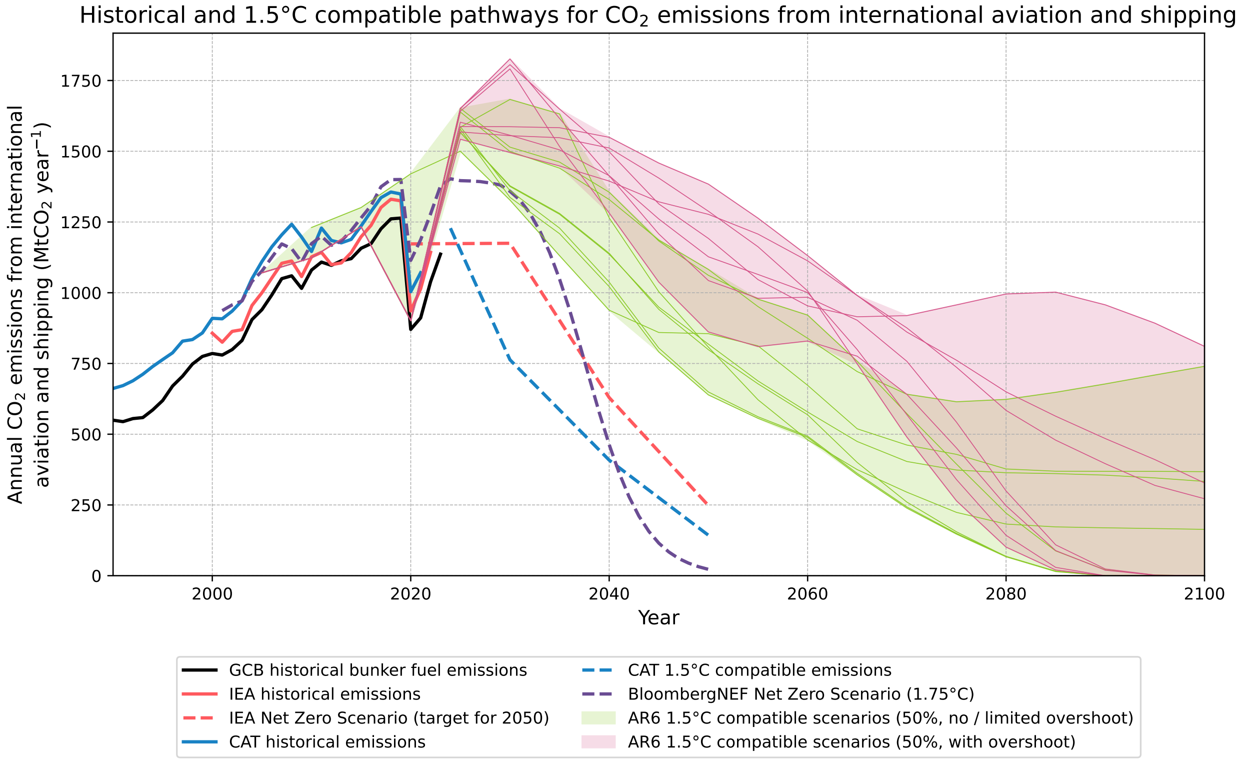

Supplementary Figure 18: **Annual CO_2 emissions from bunker fuels in assessed scenarios that are compatible with a global warming of 1.5 °C.**

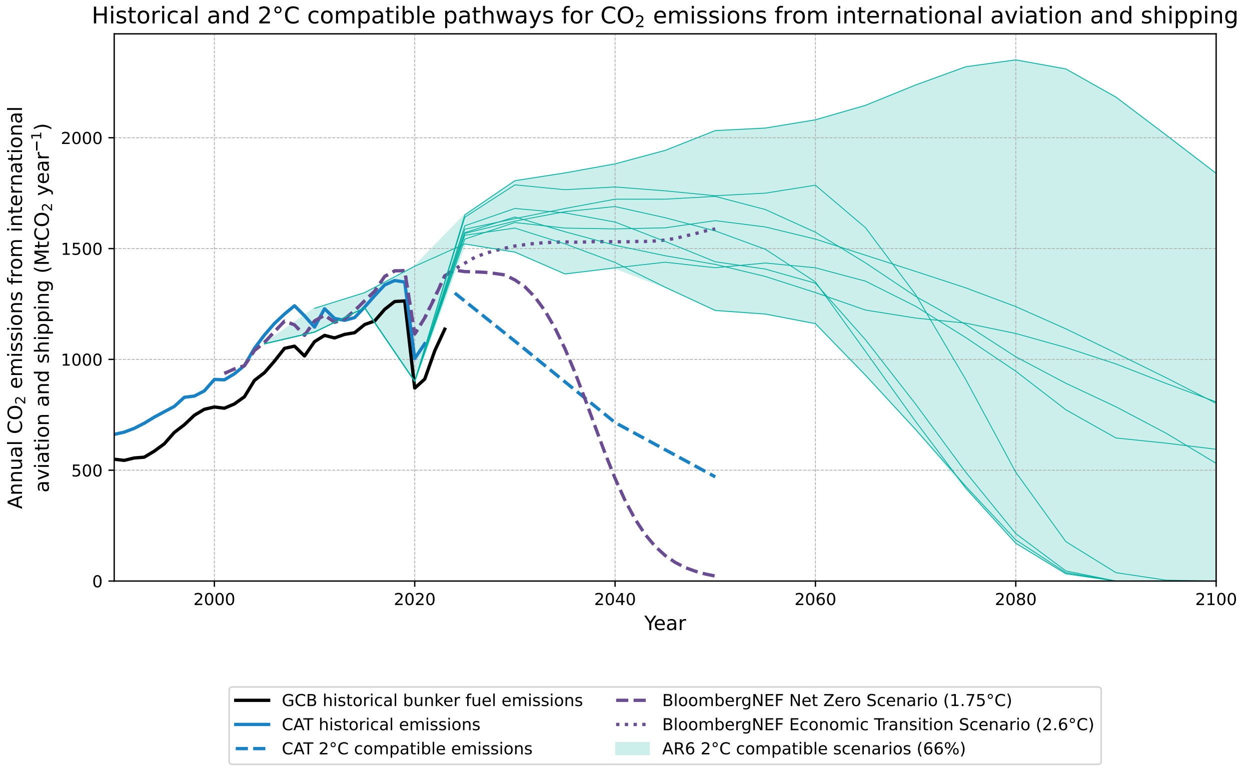

Supplementary Figure 19: **Annual CO_2 emissions from bunker fuels in assessed scenarios that are compatible with a global warming of 2 °C.**

Emission pathway implied by Switzerland's 2025 NDC

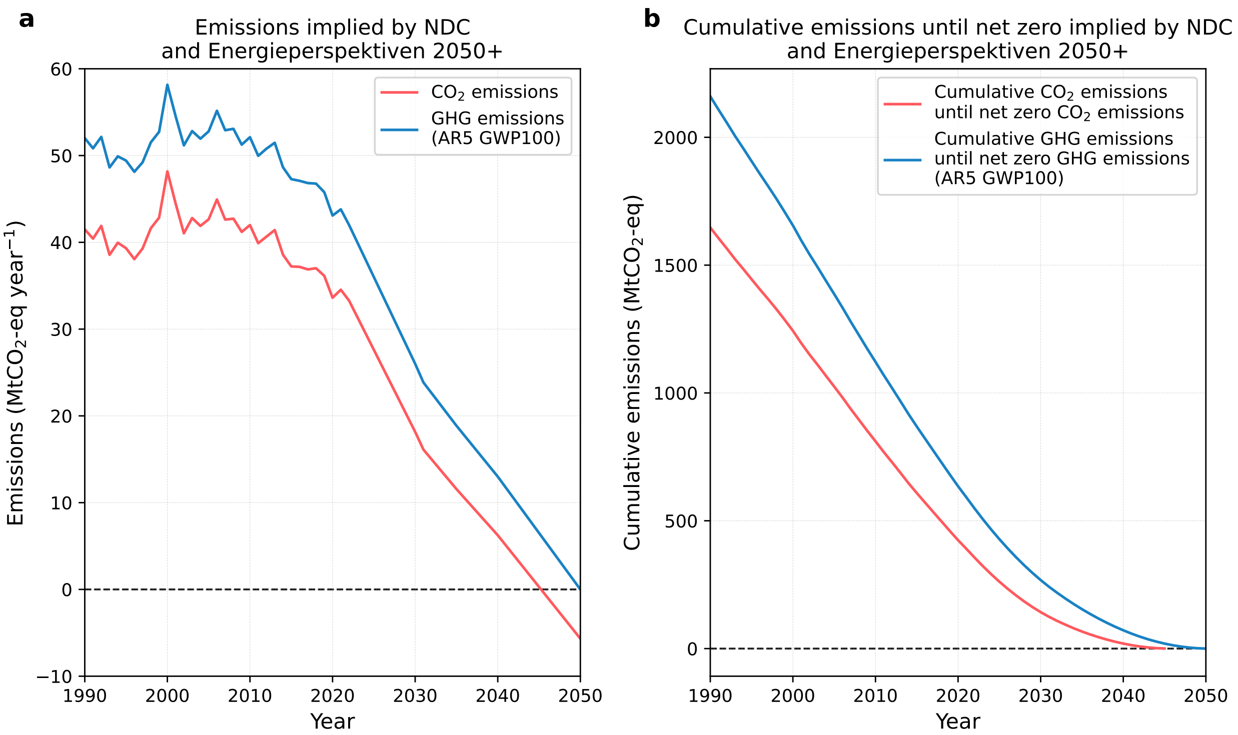

Supplementary Figure 20: **NDC-derived emission pathway for Switzerland.** **a** Switzerland CO_2 and GHG emissions from 1990 and 2050 that are consistent with the emission targets specified in Switzerland's 2025 NDC[14] and the *ZERO Basis* scenario of Switzerland's Energy Perspectives 2050+[16]. **b** Cumulative CO_2 and GHG emissions from a point in time until net zero CO_2 (2045) or net zero GHG (2050) emissions. CO_2 removal after net zero CO_2 is reached is not considered in the cumulative CO_2 emissions.

5 Additional Tables

Supplementary Table 1: **Exceedance of fair-share RCB in 2022 by UNFCCC member states.** Number of UNFCCC member states that already exceeded their fair share of the global NGHGI-consistent RCB in 2022, the population, and the fraction of GDP they represent – dependent on the temperature limit and the inclusion of CO_2 emissions from LULUCF.

**2022**			
Responsibility for CO_2 emissions	UNFCCC member states	Population (billion)	Global GDP (PPP)
	**1.5 °C (50%)**		
Including LULUCF from BMs	57–79 (29–40%)	1.6–2.3 (20–29%)	46–56%
Fossil emissions only	61–72 (31–37%)	1.6–2.0 (20–24%)	48–53%
	**2 °C (66%)**		
Including LULUCF from BMs	37–57 (19–29%)	1.2–1.6 (15–20%)	37–47%
Fossil emissions only	33–49 (17–25%)	1.1–1.5 (14–19%)	38–46%

Supplementary Table 2: **Exceedance of fair-share RCB in 2025 by UNFCCC member states.** Number of UNFCCC member states that already exceeded their fair share of the global NGHGI-consistent RCB in 2025 and the population they represent – dependent on the temperature limit, the inclusion of CO_2 emissions from LULUCF and the chosen period used to extrapolate per-capita CO_2 emissions.

**2025**			
Responsibility for CO_2 emissions	Extrap. period	UNFCCC member states	Population (billion)
	**1.5 °C (50%)**		
Including LULUCF from BMs	2013–2022	64–85 (32–43%)	1.7–2.5 (21–31%)
	2000–2022	64–87 (32–44%)	1.7–4.1 (21–50%)
Fossil emissions only	2013–2022	62–74 (31–38%)	1.6–3.4 (20–42%)
	2000–2022	62–73 (31–37%)	1.6–3.4 (20–42%)
	**2 °C (66%)**		
Including LULUCF from BMs	2013–2022	41–62 (21–31%)	1.3–1.8 (16–23%)
	2000–2022	41–62 (21–31%)	1.3–1.8 (16–23%)
Fossil emissions only	2013–2022	38–57 (19–29%)	1.2–1.6 (14–20%)
	2000–2022	38–57 (19–29%)	1.2–1.6 (14–20%)

Supplementary Table 3: **Exceedance of fair-share RCB in 2035 by UNFCCC member states.** Number of UNFCCC member states that will exceed their fair share of the global NGHGI-consistent RCB in 2035 and the population they represent – dependent on the temperature limit, the inclusion of CO_2 emissions from LULUCF and the chosen period used to extrapolate per-capita CO_2 emissions.

		**2035**	
Responsibility for CO_2 emissions	Extrap. period	UNFCCC member states	Population (billion)
		**1.5 °C (50%)**	
Including LULUCF from BMs	2013–2022	79–97 (40–49%)	3.7–4.2 (42–48%)
	2000–2022	77–103 (39–52%)	3.8–4.5 (43–51%)
Fossil emissions only	2013–2022	76–92 (39–47%)	3.2–3.7 (37–42%)
	2000–2022	82–95 (42–48%)	3.4–3.7 (39–42%)
		**2 °C (66%)**	
Including LULUCF from BMs	2013–2022	51–77 (26–39%)	1.6–2.0 (18–23%)
	2000–2022	48–75 (24–38%)	1.5–3.6 (17–41%)
Fossil emissions only	2013–2022	50–67 (25–34%)	1.4–3.1 (16–35%)
	2000–2022	51–67 (26–34%)	2.8–3.2 (32–37%)

6 Countries in datasets

This analysis relies on datasets that exclude certain Parties to the UNFCCC or report data for additional territories, that are either disputed or are located overseas, sometimes with a certain degree of autonomy. As a result, the number of country-level carbon budgets varies with the allocation approach chosen.

6.1 Equal-per-capita allocations

In addition to the parties to the UNFCCC, the population dataset used includes the following additional parties that are either only partially recognized countries, disputed territories or overseas sub-national territories, often with a certain degree of autonomy:

1. American Samoa
2. Anguilla
3. Aruba
4. Bermuda
5. Bonaire Sint Eustatius and Saba
6. British Virgin Islands
7. Cayman Islands
8. Curacao
9. Falkland Islands
10. Faroe Islands
11. French Guiana
12. French Polynesia
13. Gibraltar
14. Greenland
15. Guadeloupe
16. Guam
17. Guernsey

18. Hong Kong
19. Isle of Man
20. Jersey
21. Kosovo
22. Macao
23. Martinique
24. Mayotte
25. Montserrat
26. New Caledonia
27. Northern Mariana Islands
28. Puerto Rico
29. Reunion
30. Saint Barthelemy
31. Saint Helena
32. Saint Martin (French part)
33. Saint Pierre and Miquelon
34. Sint Maarten (Dutch part)
35. Svalbard and Jan Mayen
36. Taiwan
37. Tokelau
38. Turks and Caicos Islands
39. United States Virgin Islands
40. Wallis and Futura
41. Western Sahara

These additional parties account for 41 million people (0.5 % of the global population), with more than half of them living in Taiwan.

6.2 Territorial and consumption-based emission debts

Parties to the UNFCCC not present in fossil CO_2 emission data set of the Global Carbon Budget 2024[2].

1. San Marino
2. Holy See (governing body of the Vatican City State)
3. Monaco

As they still have reported LULUCF CO_2 emissions from bookkeeping models, we still include them, assuming that fossil CO_2 emissions are negligible.

Consumption-based CO_2 emissions are available for 118 UNFCCC member states as well as Hong Kong and Taiwan. Consumption-based allocation is therefore only performed for these countries.

6.3 Capacity-based allocation

GDP-per capita data is not available for the following Parties to the UNFCCC:

1. Andorra
2. Antigua and Barbuda
3. Bahamas
4. Belize
5. Bhutan
6. Brunei
7. Cook Islands
8. East Timor
9. Eritrea
10. Fiji
11. Grenada
12. Guyana

13. Holy See (governing body of the Vatican City State)
14. Kiribati
15. Liechtenstein
16. Maledives
17. Marshall Islands
18. Micronesia
19. Monaco
20. Nauru
21. Niue
22. Palau
23. Papua New Guinea
24. Saint Kitts and Nevis
25. Saint Vincent and the Grenadines
26. Samoa
27. San Marino
28. Solomon Islands
29. Somalia
30. South Sudan
31. Sudan
32. Suriname
33. Tonga
34. Tuvalu
35. Vanatu

For following countries or territories GDP-per-capita values are available and hence, country-level carbon budgets are calculated and provided:

1. Former Sudan (today separated in South Sudan and Sudan)
2. Hong Kong
3. Taiwan

Capacity-dependent country-level carbon budgets are therefore calculated for 165 countries.

Supplementary References

1. Gidden, M. J. *et al.* Aligning climate scenarios to emissions inventories shifts global benchmarks. en. *Nature* **624,** 102–108. ISSN: 0028-0836, 1476-4687. https://www.nature.com/articles/s41586-023-06724-y (2024) (Dec. 2023).
2. Friedlingstein, P. *et al. Global Carbon Budget 2024* en. Nov. 2024. https://essd.copernicus.org/preprints/essd-2024-519/ (2024).
3. Grassi, G. *et al. Harmonising the land-use flux estimates of global models and national inventories for 2000-2020: background data* en. Feb. 2023. https://zenodo.org/record/7541524 (2025).
4. European Commission, Joint Research Centre, EU Observatory on deforestation and forest degradation. *Global land-use carbon fluxes* 2024. https://forest-observatory.ec.europa.eu (2025).
5. Quilcaille, Y., Gasser, T., Ciais, P. & Boucher, O. CMIP6 simulations with the compact Earth system model OSCAR v3.1. en. *Geoscientific Model Development* **16,** 1129–1161. ISSN: 1991-9603. https://gmd.copernicus.org/articles/16/1129/2023/ (2025) (Feb. 2023).
6. Boeing. *Commercial Market Outlook 2024–2043* 2024. https://www.boeing.com/commercial/market/commercial-market-outlook#overview (2025).
7. Terrenoire, E., Hauglustaine, D. A., Gasser, T. & Penanhoat, O. The contribution of carbon dioxide emissions from the aviation sector to future climate change. *Environmental Research Letters* **14.** Publisher: IOP Publishing, 084019. https://dx.doi.org/10.1088/1748-9326/ab3086 (July 2019).
8. IPCC. *2006 IPCC Guidelines for National Greenhouse Gase Inventories* (eds Eggleston, S., Buendia, L., Miwa, K., Ngara, T. & Tanabe, K.) ISBN: 4-88788-032-4 (IGES, Japan, 2006).
9. Friedlingstein, P. *et al.* Global Carbon Budget 2024. en. *Earth System Science Data* **17,** 965–1039. ISSN: 1866-3516. https://essd.copernicus.org/articles/17/965/2025/ (2025) (Mar. 2025).

10. Raupach, M. R. *et al.* Sharing a quota on cumulative carbon emissions. en. *Nature Climate Change* **4,** 873–879. ISSN: 1758-678X, 1758-6798. https://www.nature.com/articles/nclimate2384 (2024) (Oct. 2014).

11. Matthews, H. D. Quantifying historical carbon and climate debts among nations. en. *Nature Climate Change* **6,** 60–64. ISSN: 1758-678X, 1758-6798. https://www.nature.com/articles/nclimate2774 (2025) (Sept. 2015).

12. Hahn, T., Morfeldt, J., Höglund, R., Karlsson, M. & Fetzer, I. Estimating countries' additional carbon accountability for closing the mitigation gap based on past and future emissions. en. *Nature Communications* **15,** 9707. ISSN: 2041-1723. https://www.nature.com/articles/s41467-024-54039-x (2024) (Nov. 2024).

13. Fanning, A. L. & Hickel, J. Compensation for atmospheric appropriation. en. *Nature Sustainability* **6,** 1077–1086. ISSN: 2398-9629. https://www.nature.com/articles/s41893-023-01130-8 (2024) (June 2023).

14. Bundesamt für Umwelt. *Switzerland's second nationally determined contribution under the Paris Agreement 2031–2035* en. 2025. https://www.bafu.admin.ch/bafu/de/home/themen/klima/fachinformationen/klima--internationales/eingaben-der-schweiz-im-rahmen-der-internationalen-klimaverhandl/eingaben-der-schweiz-im-rahmen-der-internationalen-klimaverhandlungen-unfccc-2025.html (2025).

15. United Nations Development Programme. *All composite indices and component time series (1990-2022)* https://hdr.undp.org/sites/default/files/2023-24_HDR/HDR23-24_Composite_indices_complete_time_series.csv (2024).

16. Bundesamt für Energie. *EP2050+ Senarienergebnisse ZERO Basis* Apr. 2022. https://www.bfe.admin.ch/bfe/de/home/politik/energieperspektiven-2050-plus.exturl.html/aHR0cHM6Ly9wdWJkYi5iZmUuYWRtaW4uY2gvZGUvcHVibGljYX/Rpb24vZG93bmxvYWQvMTA[illegible] .html.
