# Supplementary material for: Tracking country-level mitigation progress using NGHGI-consistent carbon budgets
Source: Nat Commun. 2026 Feb 13;17:1494. doi: 10.1038/s41467-026-69078-9 (PMC12905252; doi:10.1038/s41467-026-69078-9)
Supplement: Supplementary file 2 — Reporting Summary [file 41467_2026_69078_MOESM2_ESM.pdf]

nature portfolio

Corresponding author(s): Konstantin Weber

Last updated by author(s): Jan 6, 2026

Reporting Summary

Nature Portfolio wishes to improve the reproducibility of the work that we publish. This form provides structure for consistency and transparency in reporting. For further information on Nature Portfolio policies, see our Editorial Policies and the Editorial Policy Checklist.

Statistics

For all statistical analyses, confirm that the following items are present in the figure legend, table legend, main text, or Methods section.

n/a	Confirmed	
☐	☒	The exact sample size (*n*) for each experimental group/condition, given as a discrete number and unit of measurement
☒	☐	A statement on whether measurements were taken from distinct samples or whether the same sample was measured repeatedly
☒	☐	The statistical test(s) used AND whether they are one- or two-sided<br>*Only common tests should be described solely by name; describe more complex techniques in the Methods section.*
☒	☐	A description of all covariates tested
☒	☐	A description of any assumptions or corrections, such as tests of normality and adjustment for multiple comparisons
☒	☐	A full description of the statistical parameters including central tendency (e.g. means) or other basic estimates (e.g. regression coefficient) AND variation (e.g. standard deviation) or associated estimates of uncertainty (e.g. confidence intervals)
☒	☐	For null hypothesis testing, the test statistic (e.g. *F*, *t*, *r*) with confidence intervals, effect sizes, degrees of freedom and *P* value noted<br>*Give P values as exact values whenever suitable.*
☒	☐	For Bayesian analysis, information on the choice of priors and Markov chain Monte Carlo settings
☒	☐	For hierarchical and complex designs, identification of the appropriate level for tests and full reporting of outcomes
☒	☐	Estimates of effect sizes (e.g. Cohen's *d*, Pearson's *r*), indicating how they were calculated

Our web collection on statistics for biologists contains articles on many of the points above.

Software and code

Policy information about availability of computer code

Data collection	The data that we use comes from the following sources:<br>The global RCB from the main text and Supplementary Information of Lamboll et al., (2023): https://www.nature.com/articles/s41558-023-01848-5<br>Historical territorial and consumtion-based CO2 emissions, including LULUCF CO2 emissions estimated from bookkeeping models from the Global Carbon Budget 2024: https://www.icos-cp.eu/science-and-impact/global-carbon-budget/2024<br>Country-reported LULUCF CO2 emissions from Joint Research Centre of the European Commission: https://forest-observatory.ec.europa.eu/carbon/fluxes<br>Climate scenario data from the AR6 Scenario Database: https://data.ene.iiasa.ac.at/ar6/#/login?redirect=%2Fworkspaces<br>Correction terms from Gidden et al., 2023 available on GitHub: https://github.com/iiasa/gidden_ar6_reanalysis/tree/main<br>CO2 emissions from bunker fuels from the IEA: https://www.iea.org/data-and-statistics/charts/co2-emissions-from-international-shipping-in-the-net-zero-scenario-2000-2030-3 and https://www.iea.org/data-and-statistics/charts/co2-emissions-in-aviation-in-the-net-zero-scenario-2000-2030 and https://www.iea.org/reports/net-zero-roadmap-a-global-pathway-to-keep-the-15-0c-goal-in-reach<br>Bunker fuel emission data from Climate Action Tracker: https://climateactiontracker.org/documents/1170/202310_CAT_AssessmentData_IntShipping.xlsx and https://climateactiontracker.org/documents/1274/CAT_2024-10-23_DataAssessment_Aviation_ratingbars.xlsx<br>Population data from Our World in Data: https://ourworldindata.org/grapher/population<br>Swiss population data from the Federal Statistics Office: https://dam-api.bfs.admin.ch/hub/api/dam/assets/32229222/master<br>GDP data from the Maddison Project Database: https://dataverse.nl/citation?persistentId=doi:10.34894/INZBF2<br>Population projections from the Wittgenstein Center: https://zenodo.org/doi/10.5281/zenodo.10618931<br>Projections of future CO2 and greenhouse gas emissions for Switzerland from the Federal Office for Energy: https://www.bfe.admin.ch/bfe/

de/home/politik/energieperspektiven-2050-plus.exturl.html/aHR0cHM6Ly9wdWJkYi5iZmUuYWRtaW4uY2gvZGUvcHVibGljYX/Rpb24vZG93bmxvYWQvMTA0MzQ=.html
Planned greenhouse gas emissions for Switzerland from Switzerland's updated NDC: https://www.bafu.admin.ch/bafu/de/home/themen/klima/fachinformationen/klima--internationales/eingaben-der-schweiz-im-rahmen-der-internationalen-klimaverhandl/eingaben-der-schweiz-im-rahmen-der-internationalen-klimaverhandlungen-unfccc-2025.html

Data analysis: The code used for analysis and the creation of figures will be made publicly available upon publication in an online repository (https://doi.org/10.5281/zenodo.17426185).

For manuscripts utilizing custom algorithms or software that are central to the research but not yet described in published literature, software must be made available to editors and reviewers. We strongly encourage code deposition in a community repository (e.g. GitHub). See the Nature Portfolio guidelines for submitting code & software for further information.

Data

Policy information about availability of data

All manuscripts must include a data availability statement. This statement should provide the following information, where applicable:
- Accession codes, unique identifiers, or web links for publicly available datasets
- A description of any restrictions on data availability
- For clinical datasets or third party data, please ensure that the statement adheres to our policy

All the data underlying the figures of the main text and the Supplementary Information as well as the dataset provided are made publicly available upon publication in an online repository (https://doi.org/10.5281/zenodo.17426185).

Research involving human participants, their data, or biological material

Policy information about studies with human participants or human data. See also policy information about sex, gender (identity/presentation), and sexual orientation and race, ethnicity and racism.

Reporting on sex and gender: *Use the terms sex (biological attribute) and gender (shaped by social and cultural circumstances) carefully in order to avoid confusing both terms. Indicate if findings apply to only one sex or gender; describe whether sex and gender were considered in study design; whether sex and/or gender was determined based on self-reporting or assigned and methods used.*
Provide in the source data disaggregated sex and gender data, where this information has been collected, and if consent has been obtained for sharing of individual-level data; provide overall numbers in this Reporting Summary. Please state if this information has not been collected.
Report sex- and gender-based analyses where performed, justify reasons for lack of sex- and gender-based analysis.

Reporting on race, ethnicity, or other socially relevant groupings: *Please specify the socially constructed or socially relevant categorization variable(s) used in your manuscript and explain why they were used. Please note that such variables should not be used as proxies for other socially constructed/relevant variables (for example, race or ethnicity should not be used as a proxy for socioeconomic status).*
Provide clear definitions of the relevant terms used, how they were provided (by the participants/respondents, the researchers, or third parties), and the method(s) used to classify people into the different categories (e.g. self-report, census or administrative data, social media data, etc.)
Please provide details about how you controlled for confounding variables in your analyses.

Population characteristics: *Describe the covariate-relevant population characteristics of the human research participants (e.g. age, genotypic information, past and current diagnosis and treatment categories). If you filled out the behavioural & social sciences study design questions and have nothing to add here, write "See above."*

Recruitment: *Describe how participants were recruited. Outline any potential self-selection bias or other biases that may be present and how these are likely to impact results.*

Ethics oversight: *Identify the organization(s) that approved the study protocol.*

Note that full information on the approval of the study protocol must also be provided in the manuscript.

Field-specific reporting

Please select the one below that is the best fit for your research. If you are not sure, read the appropriate sections before making your selection.

☐ Life sciences ☐ Behavioural & social sciences ☒ Ecological, evolutionary & environmental sciences

For a reference copy of the document with all sections, see nature.com/documents/nr-reporting-summary-flat.pdf

Ecological, evolutionary & environmental sciences study design

All studies must disclose on these points even when the disclosure is negative.

Study description: We align the size of the global RCB that is distributable to countries with national CO2 accounting to make the it comparable to the climate targets countries communicate (also in their NDCs). We also calculate national RCBs based on the corrected global RCB with allocation approaches found in previous literature. This allows an assessment of how many and which countries have already

	exceeded their national RCB (according to a specific allocation approach and a specific temperature limit and likelihood).
Research sample	Part of the study focuses on a global quantity. For allocations of the global remaining carbon budget to countries we perform calculations for all countries were data is available, which is dependent on the allocation approach (see Methods and Supplementary Information). For certain statements we limit ourselves to countries (and their population / GDP) that are part of the UNFCCC.
Sampling strategy	For the correction terms we propose, we take mean values of relevant scenario categories (see Methods). For the 1.5 °C (50%) RCB and the 2 °C (66%) RCB we use the mean of C1 and C3 scenarios, respectively, that were reanalyzed by Gidden et al., 2023. Similarly, for the correction related to bunker fuels, we use the mean of C1 and C3 scenarios with available data in the AR6 scenario database.
Data collection	The data sources we rely on are publicly available. They are either part of the peer-reviewed literature or are provided by official bodies of countries.
Timing and spatial scale	For our global analysis, we focus on 1990 to 2024 (due to data availability, mainly country-reported emissions from NGHGIs). National RCBs are provided back to 1990 and dependent on the allocation method and the necessary data, national RCBs can be calculated until 2022 or 2023. We also extrapolate national RCBs until 2035 based on a linear extrapolation of the year-to-year change in the per-capita national RCB between 2013-2022. To calculate historical responsibility of countries for some allocation approaches, we use emissions data from the Global Carbon Budget 2024 that goes back until 1850. The analysis extends to all countries globally with available data.
Data exclusions	Countries with missing data are excluded, but calculations are always performed for the biggest subset of countries with available data (see Supplementary Information and national RCB data available upon publication).
Reproducibility	The formulas used for calculations are documented in the manuscript and the code to reproduce the figures and results will publicly available once the manuscript is published. The data used is publicly available.
Randomization	Not applicable.
Blinding	Not applicable.

Did the study involve field work? ☐ Yes ☒ No

Reporting for specific materials, systems and methods

We require information from authors about some types of materials, experimental systems and methods used in many studies. Here, indicate whether each material, system or method listed is relevant to your study. If you are not sure if a list item applies to your research, read the appropriate section before selecting a response.

Materials & experimental systems

n/a	Involved in the study	
☒	☐	Antibodies
☒	☐	Eukaryotic cell lines
☒	☐	Palaeontology and archaeology
☒	☐	Animals and other organisms
☒	☐	Clinical data
☒	☐	Dual use research of concern
☒	☐	Plants

Methods

n/a	Involved in the study	
☒	☐	ChIP-seq
☒	☐	Flow cytometry
☒	☐	MRI-based neuroimaging

Plants

Seed stocks	*Report on the source of all seed stocks or other plant material used. If applicable, state the seed stock centre and catalogue number. If plant specimens were collected from the field, describe the collection location, date and sampling procedures.*
Novel plant genotypes	*Describe the methods by which all novel plant genotypes were produced. This includes those generated by transgenic approaches, gene editing, chemical/radiation-based mutagenesis and hybridization. For transgenic lines, describe the transformation method, the number of independent lines analyzed and the generation upon which experiments were performed. For gene-edited lines, describe the editor used, the endogenous sequence targeted for editing, the targeting guide RNA sequence (if applicable) and how the editor was applied.*
Authentication	*Describe any authentication procedures for each seed stock used or novel genotype generated. Describe any experiments used to assess the effect of a mutation and, where applicable, how potential secondary effects (e.g. second site T-DNA insertions, mosiacism, off-target gene editing) were examined.*
